# Association of MicroRNAs With Risk of Stroke: A Meta-Analysis

**DOI:** 10.3389/fneur.2022.865265

**Published:** 2022-05-19

**Authors:** Yidong Deng, Peijian Huang, Fan Zhang, Tao Chen

**Affiliations:** Hainan General Hospital, Hainan Affiliated Hospital of Hainan Medical University, Haikou, China

**Keywords:** microRNA, ischemic stroke, sensitivity, diagnostic odds ratio, meta-analysis

## Abstract

**Objectives:**

Altered expression of microRNAs (miRNAs) may contribute to disease vulnerability. Studies have reported the involvement of miRNA in the pathophysiology of ischemic stroke.

**Methods:**

We performed a meta-analysis of data from 6 studies that used a panel of miRNAs with altered expressions to diagnose ischemic stroke with the Bayesian framework. The *I*^2^ test and Cochran's Q-statistic were used to assess heterogeneity. Funnel plots were generated and publication bias was assessed using Begg and Egger tests.

**Results:**

On summary receiver operating characteristics (SROC) curve analysis, the pooled sensitivity and specificity of altered miRNA expressions for diagnosis of ischemic stroke was 0.92 (95% confidence interval [CI] 0.80–0.97) and 0.83 (95% CI 0.71–0.90), respectively; the diagnostic odds ratio was 54.35 (95% CI 20.39–144.92), and the area under the SROC curve was 0.93 (95% CI 0.90–0.95).

**Conclusions:**

Our results showed a link between dysregulation of miRNAs and the occurrence of ischemic stroke. Abnormal miRNA expression may be a potential biomarker for ischemic stroke.

## Introduction

Stroke is the second leading cause of death among people aged >60 years. An estimated 15 million new cases of stroke are reported each year globally, and it is one of the leading causes of death and disability among adults (1). Stroke imposes a considerable burden on the affected families and the society at large (2). Stroke is a complex neurological disorder and both hereditary and environmental factors have been implicated in its causation. Epidemiological studies have suggested a key role of genetic factors in the occurrence of stroke (3, 4).

MicroRNAs (miRNAs) are a class of endogenous ~22-nucleotide non-coding RNAs with highly conserved sequences, which can inhibit protein translation or directly lead to its degradation by specifically binding to the 3'-untranslated regions (3'-UTR) of target gene mRNAs, regulate the expression of proteins at the post-transcriptional level, and participate in a variety of pathophysiological processes (5, 6). Dysregulation of miRNAs leading to altered expression and function of proteins has been implicated in the causation of a variety of diseases. Emerging evidence suggests a potential role of miRNAs in ischemic stroke-related physiologic processes such as atherosclerosis and hypertension (7–9).

MicroRNAs play an important role in physiological and pathological processes such as cell proliferation, hematopoiesis, metabolism, immunological function, and epigenetics, in the context of cancer, neurodegenerative disorders, and other diseases (1, 10–12). Recent studies have indicated that circulating miRNAs may be novel biomarkers of cardiovascular diseases, including hypertension, stroke, diabetes, coronary artery disease, and heart failure (9, 13–16). In recent years, several studies have investigated the relation between miRNAs gene polymorphisms and stroke susceptibility (17–21); however, the findings were inconsistent because of differences in the study population and regional differences. Studies have also shown that miRNAs are associated with susceptibility to ischemic stroke, but the findings reported so far are not consistent (17, 22, 23). Therefore, we performed a meta-analysis of studies that investigated the relation between dysregulation of miRNA expression and stroke. The objective was to assess the association between miRNAs expression and stroke occurrence and to clarify the potential significance of genetic factors in the diagnosis of stroke.

## Methods

### Literature Search

The following English language electronic bibliographic databases were searched for relevant published studies: The Cochrane Library (Cochrane Database of Systematic Reviews), EMBASE, Pubmed/MEDLINE, Web of Science (science and social science citation index), Health Technology Assessment Database, and Cochrane Central Register of Controlled Trials. Moreover, the Chinese databases such as China National Knowledge Infrastructure, Technology of Chongqing VIP database, Wan Fang Data, and SinoMed were also searched.

The searched terms were related to or described the relation of miRNAs with stroke. The search terms for literature retrieval were “stroke” and “microRNAs” related terms with appropriate adjustments according to the database.

### Inclusion Criteria

In this study, all quantitative studies that explored the diagnostic value of miRNA expression for stroke were included.

Inclusion criteria: (1) study design: case-control study; (2) study population: patients with ischemic stroke diagnosed based on clinical manifestations, brain CT and/or MRI; (3) minimum sample size: >30 cases; (4) use of similar study methods, diagnostic criteria, and selection criteria for the control group; (5) matching of the control group with cases with respect to general characteristics such as race, age, and sex; (6) miRNA detection methods included quantitative PCR, RNA chip, or *in situ* hybridization.

### Data Extraction and Quality Evaluation

Two reviewers independently reviewed the included papers and extracted the data using a standardized format. The authors of the original studies were contacted in case any aspect of the study was ambiguous. Disagreements between the two reviewers, if any, were discussed and resolved by consensus or with the help of a third investigator. Data pertaining to the following variables were included: first author's name, year of publication, nation, ethnicity, age, control group source, and research sample size.

### Quality Evaluation of the Included Studies

The quality of the included studies was systematically evaluated according to QUADAS-2 guidelines. The selection of research subjects, comparability, and assessment of exposure variables were all major considerations (24). Studies with scores over 5 were considered high-quality studies, while those below 5 were considered low-quality studies. Two reviewers separately assessed the quality of the studies, and any differences in judgment were resolved following discussion with a third reviewer.

### Statistical Analysis

Using the R3.2.3 program, a meta-analysis was performed using the Bayesian framework. The *I*^2^ test and Cochran's Q-statistic were used to assess heterogeneity among the included studies and the strength of the association between miRNA levels and ischemic stroke risk was calculated using the odds ratio (OR) and 95 percent confidence interval (CI). Funnel plots were generated, and publication bias was assessed using Begg and Egger regression. *P* < 0.05 were considered indicative of statistical significance.

## Results

### Characteristics of the Included Studies

**Figure 1:**
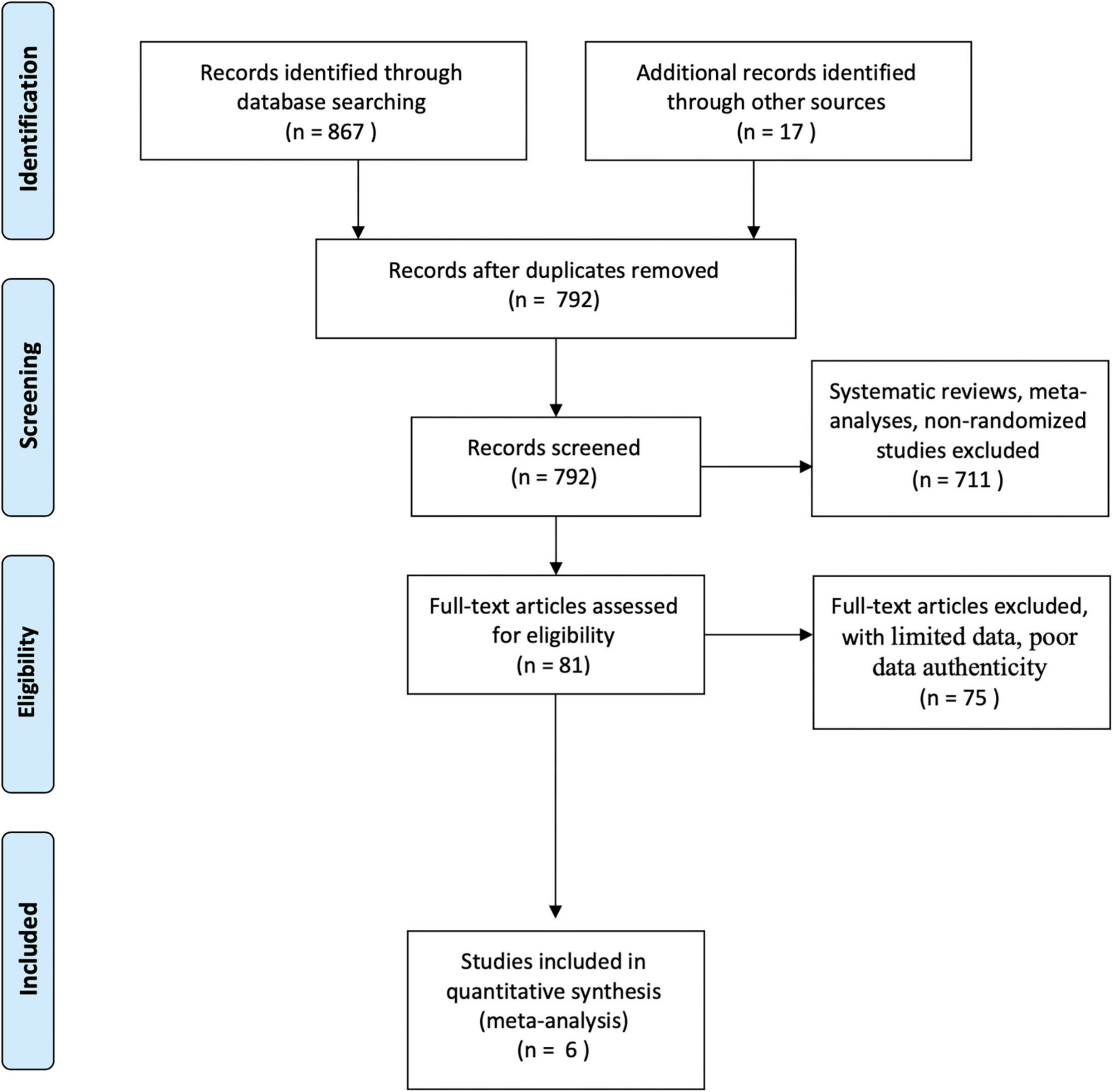
Schematic illustration of the study selection process.

A total of six studies that investigated miRNAs expression for diagnosis of stroke were included in the meta-analysis. A schematic illustration of the literature search and the study-selection criteria is presented in Figure 1.

A total of 884 studies were retrieved on database search of which 92 were excluded due to duplication. After reviewing the title and abstracts of the remaining 792 research articles, 711 were excluded. After full-text review of the remaining articles, 6 studies were found to qualify the inclusion criteria and were included in the meta-analysis (18–21, 25, 26). The combined study population of these six studies comprised of 974 patients with ischemic stroke and 679 controls. The characteristics of these studies along with the respective QUADAS scores are summarized in Table 1.

**Table 1:** Summary of included studies.

**Study**	**Year**	**Patients**	**Controls**	**Country**	**QUADAS**	**Specimen**	**MiRNAs**
Long et al. (18)	2013	197	50	China	6	Plasma	miR-30a, miR-126 and let-7b
Wu et al. (26)	2015	106	120	China	7	Serum	miR-15a, miR-16, and miR-17-5p
Huang et al. (20)	2016	302	302	China	7	Whole blood	let-7e-5p
Yang et al. (25)	2016	114	58	China	5	Plasma	miR-107, miR-128b and miR-153
Zhao et al. (19)	2016	168	104	China	6	Plasma	miR-335
Sonoda et al. (21)	2019	87	45	Japan	5	Serum	miR-1268b, miR-4433b-3p, and miR-6803-5p

### Heterogeneity Analysis and Diagnostic Value of MiRNAs

**Figure 2:**
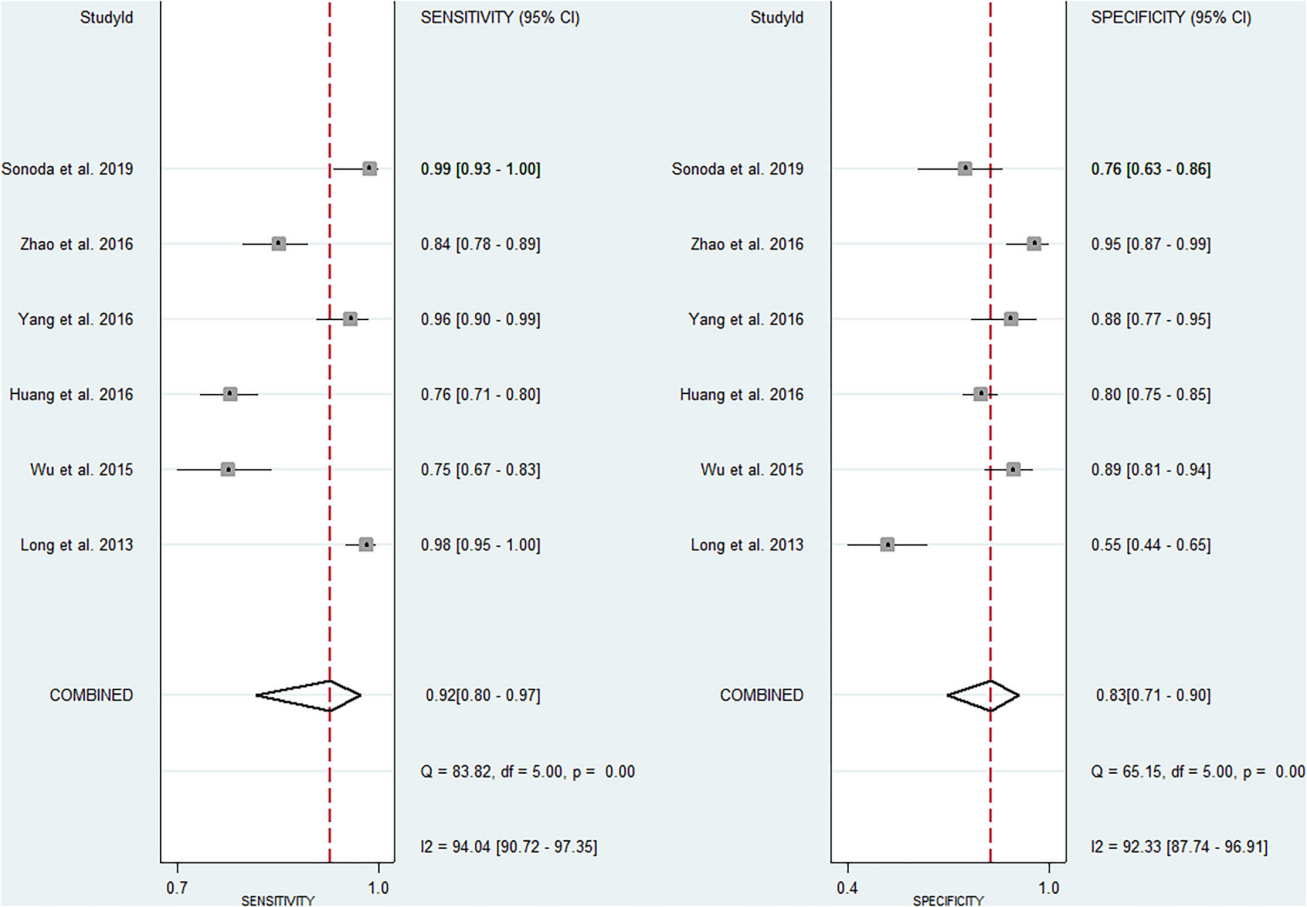
Forrest plot of estimates of sensitivity and specificity.

**Figure 3:**
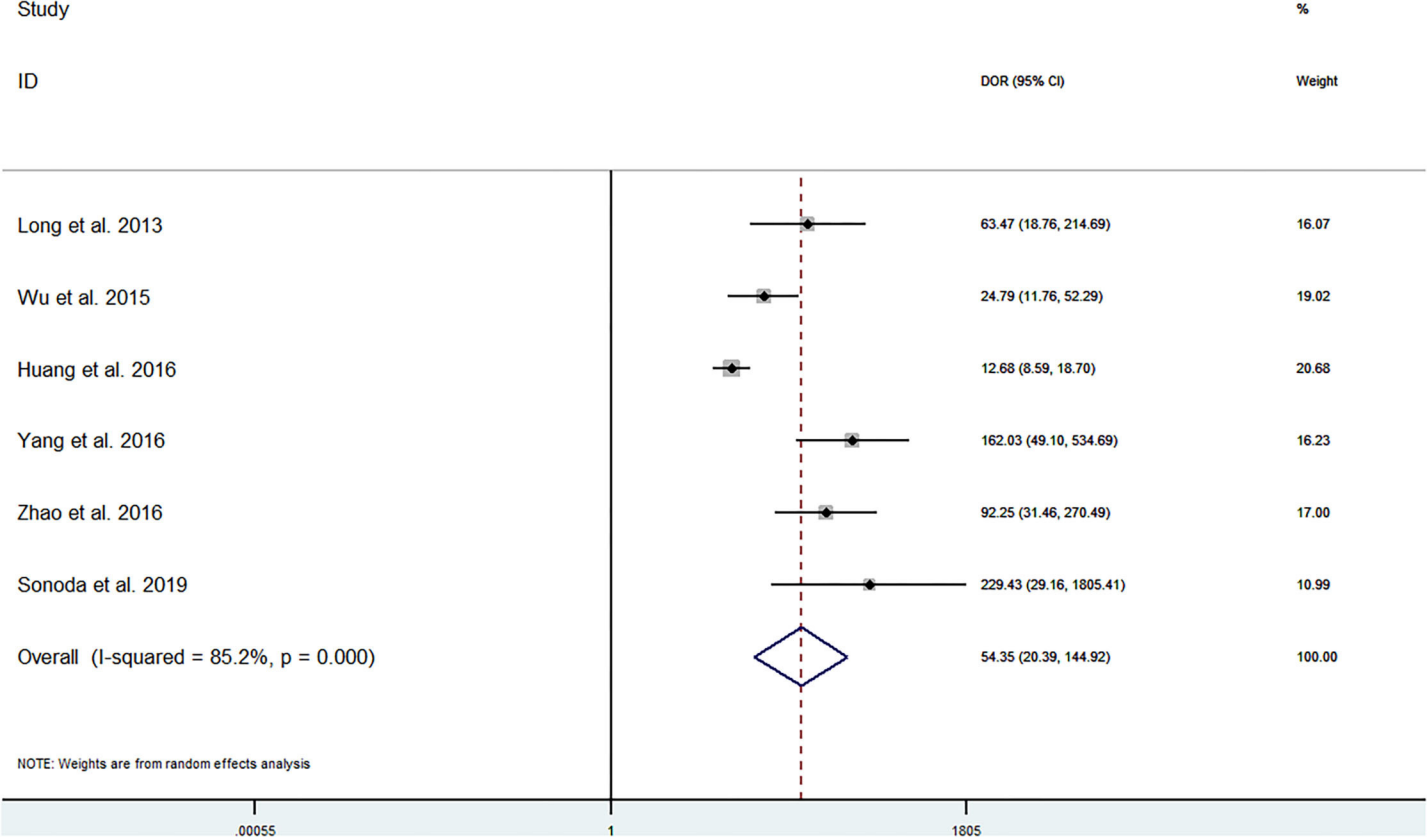
Forrest plot of diagnostic odds ratio (DOR).

**Figure 4:**
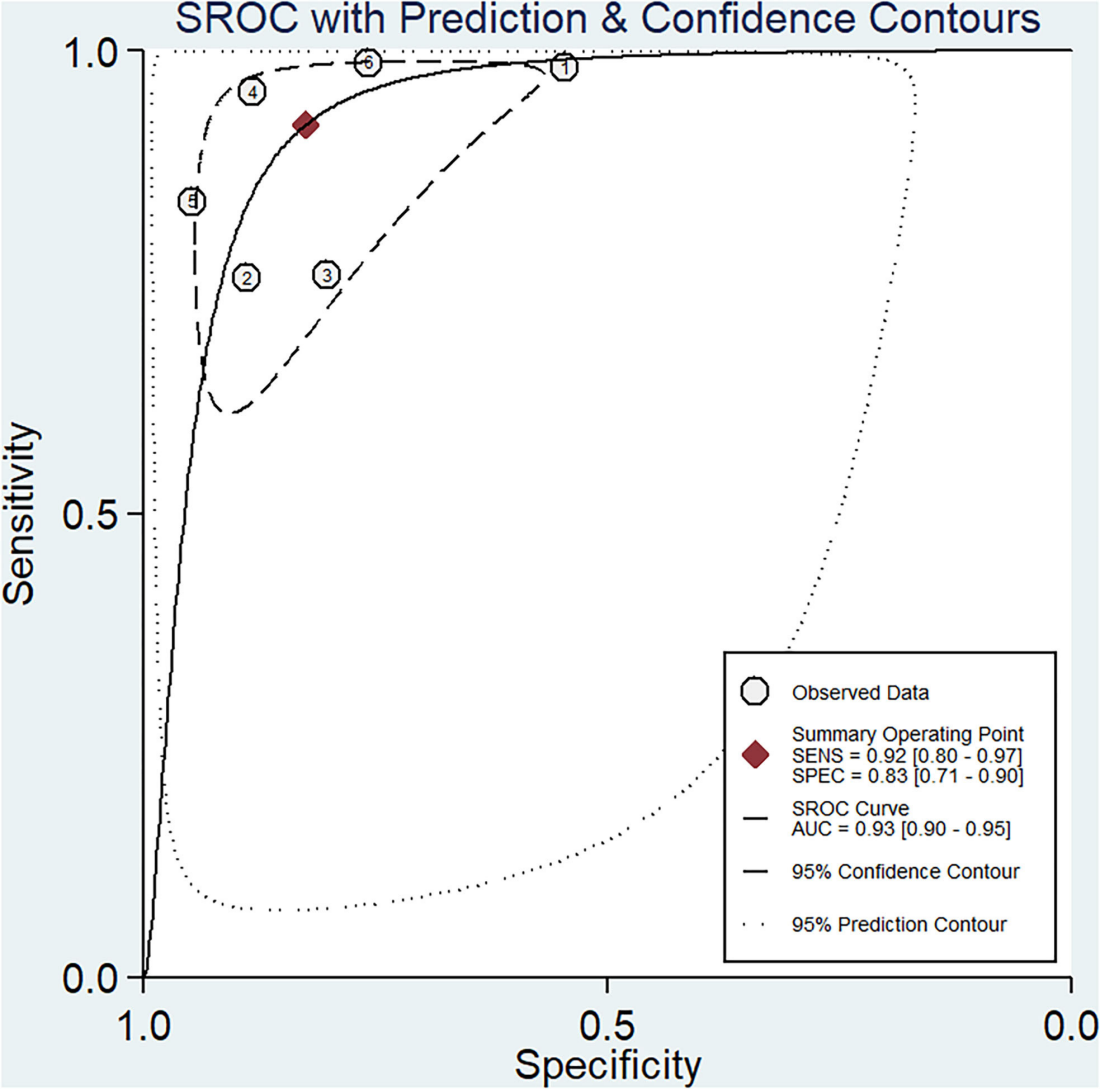
Summary receiver operating characteristic (SROC) curve.

**Figure 5:**
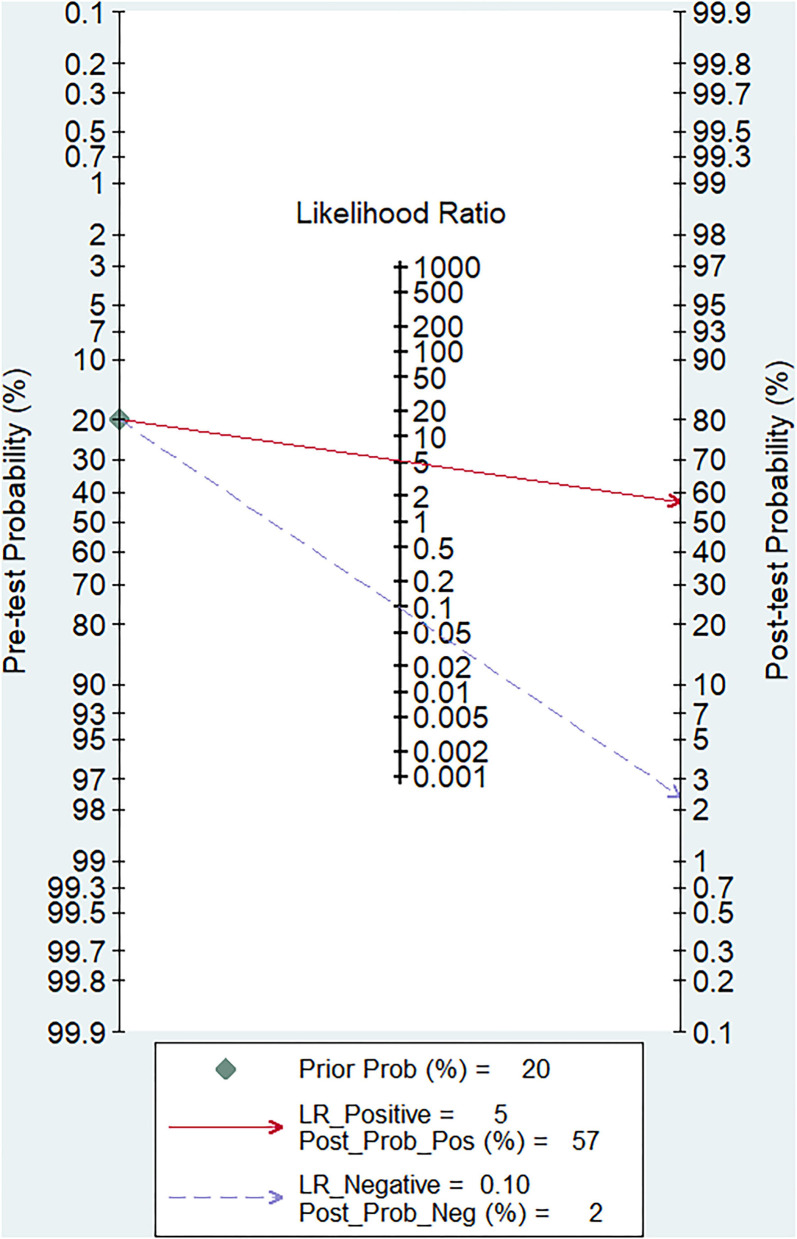
Fagan's Nomogram for assessment of post-test probabilities.

The pooled sensitivity and specificity of miRNAs for the diagnosis of ischemic stroke were 0.92 (95% CI 0.80–0.97) and 0.83 (95% CI 0.71–0.90), respectively (Figure 2). The diagnostic odds ratio was 54.35 (95% CI 20.39–144.92; Figure 3). The pooled diagnostic value of miRNAs was assessed using summary receiver operative characteristic (SROC) curve analysis; the area under the SROC was 0.93 (95% CI 0.90–0.95; Figure 4). Notably, a 3-miRNA combination model (miR-1268b, miR-4433b-3p, and miR-6803-5p) reported by Sonoda et al. showed highest sensitivity (0.99, 95% CI 0.93–1; Figure 2) and diagnostic value (229.43, 95% CI 29.16–1805.41; Figure 3) suggesting the potential role of a 3-miRNA combination model as biomarker for ischemic stroke. Additionally, as shown in the Fagan's nomogram in Figure 5, the positive likelihood ratio (PLR) was 5.3 (95% CI 3.2–8.8) and the negative likelihood ratio (NLR) was 0.10 (95% CI 0.04–0.24).

### Diagnostic Value of MiRNAs Changes in Different Stages of Ischemic Stroke

**Figure 6:**
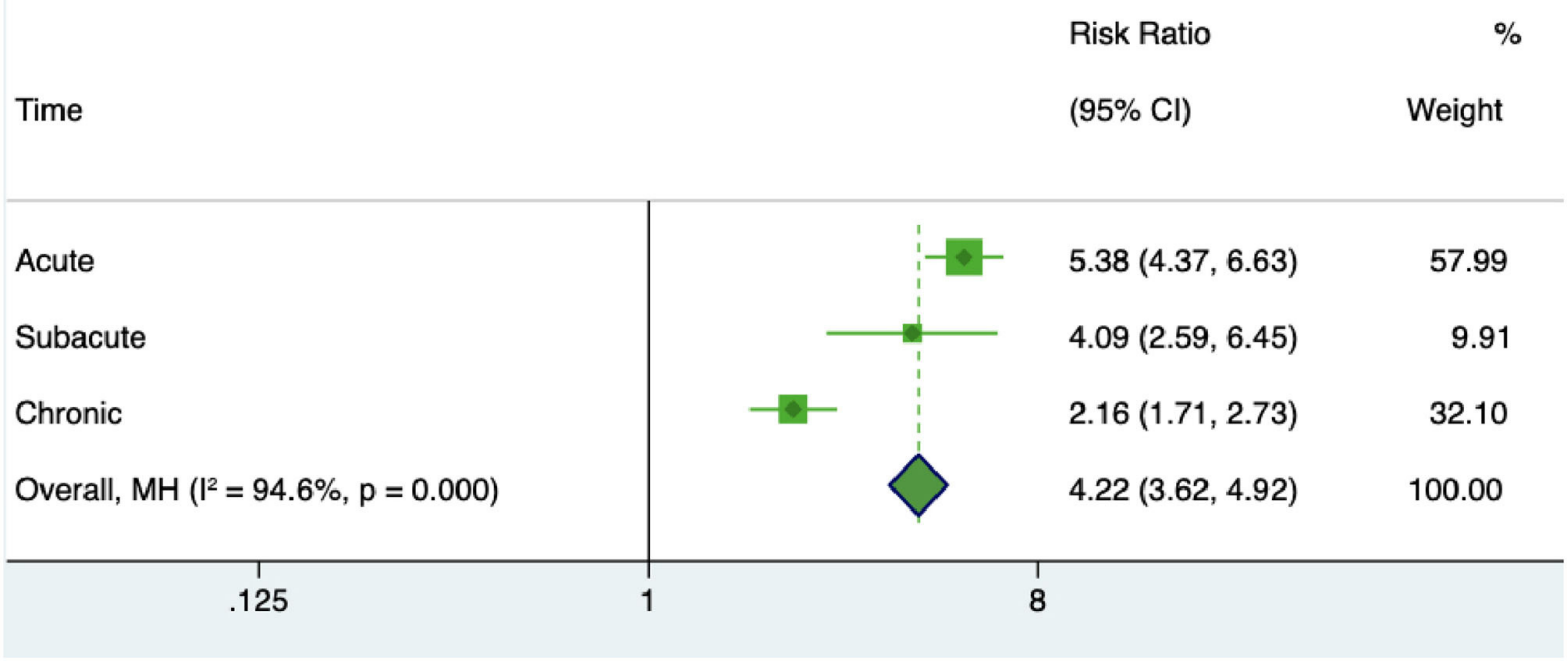
Forrest plot of risk ratio of miRNAs in different stages of stroke.

To specify the miRNA changes in different stages of stroke, we analyzed the miRNA dysregulations in acute, subacute, chronic stages of stroke. The risk ratio of miRNA in acute ischemic stroke patients was 5.38 (95% CI 4.37–6.63), in subacute stage of ischemic stroke was 4.09 (95% CI 2.56–6.45), and in chronic stage of ischemic stroke was 2.16 (95% CI 1.71–2.73) (Figure 6). These results suggested a better diagnostic value of miRNA changes in the early stages of stroke.

### Publication Bias

**Figure 7:**
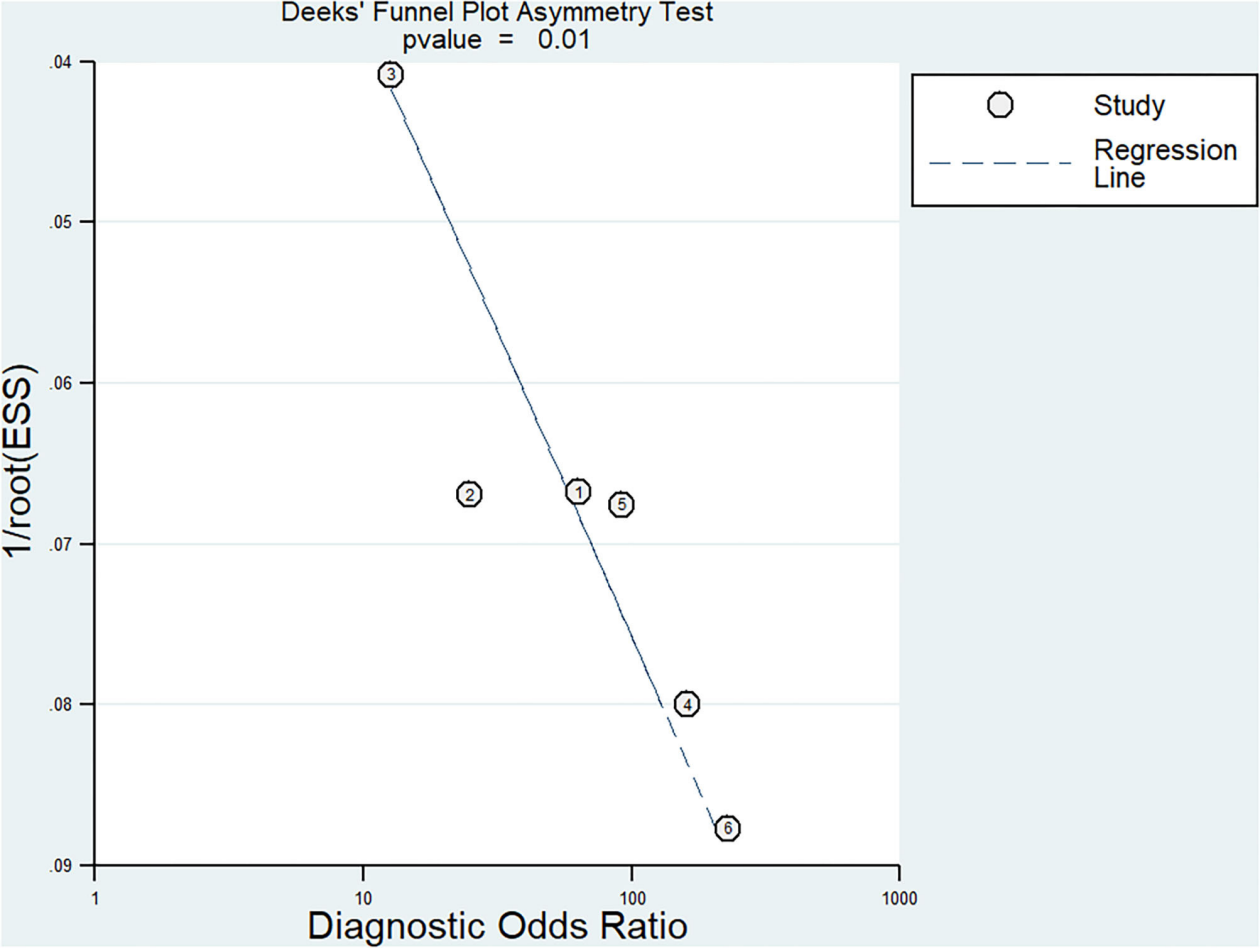
Summary receiver operating characteristic curve.

The Begg's funnel plot was used to assess the potential effect of publication bias on the results of the meta-analysis. The Deek's test of funnel plot asymmetry was used with a quantitative method to further evaluate the effect of potential publication bias. The slope coefficient was presented with a *P* value of <0.01 in overall studies, suggesting a low likelihood of publication bias (Figure 7).

## Discussion

Numerous studies have investigated the potential role of miRNAs as a biomarker for ischemic stroke. In the present study, we firstly used the pooled analysis with previously published studies and confirmed the relationship between dysregulation of miRNAs and the occurrence of ischemic stroke with quantitative methods. Additionally, we compared the diagnostic value of miRNAs in different studies and found that a 3-miRNA combination model (miR-1268b, miR-4433b-3p, and miR-6803-5p) reported by Sonoda et al. showed highest sensitivity (0.99, 95% CI 0.93–1; Figure 2) and diagnostic value (229.43, 95% CI 29.16–1805.41; Figure 3) suggesting the potential role of a 3-miRNA combination model as biomarker for ischemic stroke. The results of this meta-analysis showed a significant association between miRNAs expression dysregulation and ischemic stroke, which support the potential role of miRNAs as a diagnostic marker.

Inflammatory response plays an important role in the pathogenesis of ischemic stroke. miR-199a-5p can regulate the inflammatory response by targeting the expression of tumor necrosis factor-α, which is closely related to the development of ischemic stroke, and inhibit inflammatory mitogen-activated protein kinase pathway and nuclear factor-κB pathway (27, 28). Moreover, miRNAs (including miR-122,let-7 and miR-146a) can regulate Toll-like receptor signaling pathways and cytokines, and play a neuroprotective role through negative feedback regulatory loop regulation including IL-1 receptor-associated kinase 1 and TNF receptor-associated factor 6 (TRAF6) (29–31). Dysregulation of miRNAs may affect the expression of target mRNAs to mature mRNAs, thereby affecting the occurrence and development of diseases (32–34). Studies have shown that miR-146aG allele can lead to decreased mature miR-146a levels, and that miR-146aC polymorphism can affect the expression levels of miR-146a, IRAK-1, and TRAF6, which is associated with the risk of coronary heart disease (35). In addition, miRNAs have also been implicated in enhancing susceptibility to tumors and hypertension, and their relationship with ischemic stroke has also been studied. Therefore, we sought to draw objective conclusions on the relationship between the two through a comprehensive literature search and pooled analysis.

Numerous studies have investigated the potential role of miRNAs as biomarker for ischemic stroke patients. Chen et al. revealed that miR-146b was significantly increased within 24 h after stroke, as compared with healthy individuals, and showed a positive correlation with inflammatory response (36). Another study found that let-7e-5p level in blood was positively associated with risk of ischemic stroke and was significantly higher in patients with ischemic stroke compared to the healthy controls (20). In this meta-analysis with a large sample size, the area under the SROC curve was 0.93 (95% CI 0.90–0.95), which supports a role for miRNAs as a diagnostic tool. Pooled analysis showed a significant association of miRNAs with the risk of ischemic stroke in the overall population; however, subgroup analysis disaggregated by TOAST (Trial of Org 10 172 in acute stroke treatment) subtype was not performed due to inadequate sample size. In contrast, Jeon et al. found a significant association between miRNAs and risk of stroke in both large-artery atherosclerosis and small cerebrovascular occlusion subtypes. The results in the above studies may be attributable to different ethnicity, sample sizes, experimental designs, and stroke subtype categories Moreover, further studies are required to assess the association between miRNA dysregulation and ischemic stroke subtypes.

Although this meta-analysis revealed a link between miRNAs dysregulation and the risk of ischemic stroke, some limitations of our study should be acknowledged. First, the included studies only involved univariate analysis, while multivariate analysis provides more robust evidence of correlation in the context of complex diseases. Second, the sample size of included studies was relatively small and information about TOAST subtypes was not available for some studies. Third, both genetic and environmental factors have been implicated in the causation of stroke; however, none of the included studies assessed gene-gene or gene-environment interactions. Finally, the included studies did not account for the effect of potential confounding factors in the analysis. Therefore, larger, multicenter studies in different ethnic populations are required to determine the association between miRNAs and ischemic stroke, along with subtype analysis, haplotype analysis, ethnicity, gene-gene, and gene-environment interactions. Further, the underlying mechanisms by which genetic function changes caused by miRNAs dysregulation influence the onset and progression of ischemic stroke need to be investigated (37, 38).

## Conclusions

In conclusion, the present study suggests a potential diagnostic role of miRNAs in ischemic stroke. Compared with the standard tools such as MRI, miRNAs may not be ideal candidates for clinical use at present with satisfactory sensitivity and specificity (39). Further large-scale prospective studies should be conducted to validate the potential clinical applicability in stroke diagnosis.

## Data Availability Statement

The original contributions presented in the study are included in the article/supplementary material, further inquiries can be directed to the corresponding author/s.

## Author Contributions

YD and PH conceived and designed this research and performed statistical analysis. Data collection and extraction was performed by YD and FZ. Data was verified by YD. Statistical analysis was performed by YD and PH. YD and TC participated in drafting article. All authors read and approved the final manuscript.

## Conflict of Interest

The authors declare that the research was conducted in the absence of any commercial or financial relationships that could be construed as a potential conflict of interest.

## Publisher's Note

All claims expressed in this article are solely those of the authors and do not necessarily represent those of their affiliated organizations, or those of the publisher, the editors and the reviewers. Any product that may be evaluated in this article, or claim that may be made by its manufacturer, is not guaranteed or endorsed by the publisher.
